# ESR Essentials: diagnosis of hepatocellular carcinoma—practice recommendations by ESGAR

**DOI:** 10.1007/s00330-024-10606-w

**Published:** 2024-02-21

**Authors:** Roberto Cannella, Marc Zins, Giuseppe Brancatelli

**Affiliations:** 1 Section of Radiology - Department of Biomedicine, Neuroscience and Advanced Diagnostics (BiND), University of Palermo, Palermo, Italy; 2 Department of Radiology, Saint Joseph and Marie Lannelongue Hospitals, Paris, France

**Keywords:** Carcinoma (hepatocellular), Liver neoplasm, Liver cirrhosis, Magnetic resonance imaging, Tomography (x-ray computed)

## Abstract

**Abstract:**

Hepatocellular carcinoma (HCC) is the most common primary hepatic malignancy and a leading cause of cancer related death worldwide. Current guidelines for the noninvasive diagnosis of HCC are provided by the European Association for the Study of the Liver (EASL), the American Association for the Study of Liver Diseases (AASLD) which endorsed the Liver Imaging Reporting and Data System (LI-RADS) algorithm, the Korean Liver Cancer Association-National Cancer Center (KLCA-NCC), and the Asian-Pacific Association for the Study of the Liver (APASL). These allow the diagnosis of HCC in high-risk patients in the presence of typical imaging features on contrast-enhanced CT, MRI, or contrast-enhanced ultrasound. Size, non-rim arterial phase hyperenhancement, non-peripheral washout, enhancing capsule, and growth are major imaging features and they should be combined for the diagnosis of HCC. This article provides concise and relevant practice recommendations aimed at general radiologist audience, summarizing the best practice and informing on the essential imaging criteria for the diagnosis of HCC, while also discussing the high-risk population criteria, imaging modalities, and imaging features according to the current guidelines.

**Key Points:**

• *Noninvasive diagnosis of hepatocellular carcinoma (HCC) can be provided only in patients at high risk.*

• *Contrast-enhanced CT or MRI are the first-line imaging exams for the diagnosis of HCC.*

• *Major imaging features should be combined to provide the diagnosis of definitive HCC.*

## Key recommendations

• Noninvasive diagnosis of hepatocellular carcinoma (HCC) can be provided only in patients at high risk, including patients with cirrhosis, chronic viral hepatitis B, and current or prior HCC history (level of evidence: moderate).

• Contrast-enhanced CT or MRI are the recommended imaging exams for the diagnosis of HCC (level of evidence: high). The choice of the imaging exams and type of contrast agent should consider the availability, expertise, and patient characteristics. Contrast-enhanced ultrasound can be considered a problem-solving technique in experienced centers (level of evidence: moderate).

• The key imaging criteria for the diagnosis of HCC are size, non-rim arterial phase hyperenhancement, non-peripheral washout, enhancing capsule, and threshold growth (level of evidence: high). Hepatobiliary phase hypointensity can increase the sensitivity for the diagnosis of HCC, but it reduces the specificity as several non-HCC lesions can demonstrate hepatobiliary phase hypointensity (level of evidence: moderate). Lesions with targetoid appearance are not typical for HCC, and the diagnosis of non-HCC malignancy should be considered (level of evidence: high).

## Introduction

Liver cancer is the third most common cause of cancer-related death worldwide [1]. Hepatocellular carcinoma (HCC) is the most common primary liver carcinoma, accounting for 75% of all primary liver malignancies [2]. Up to 90% of HCCs are diagnosed in patients with cirrhosis or chronic liver disease with the main risk factors being chronic hepatitis B, chronic hepatitis C, nonalcoholic fatty liver disease, and alcohol abuse [3].

**Fig. 1:**
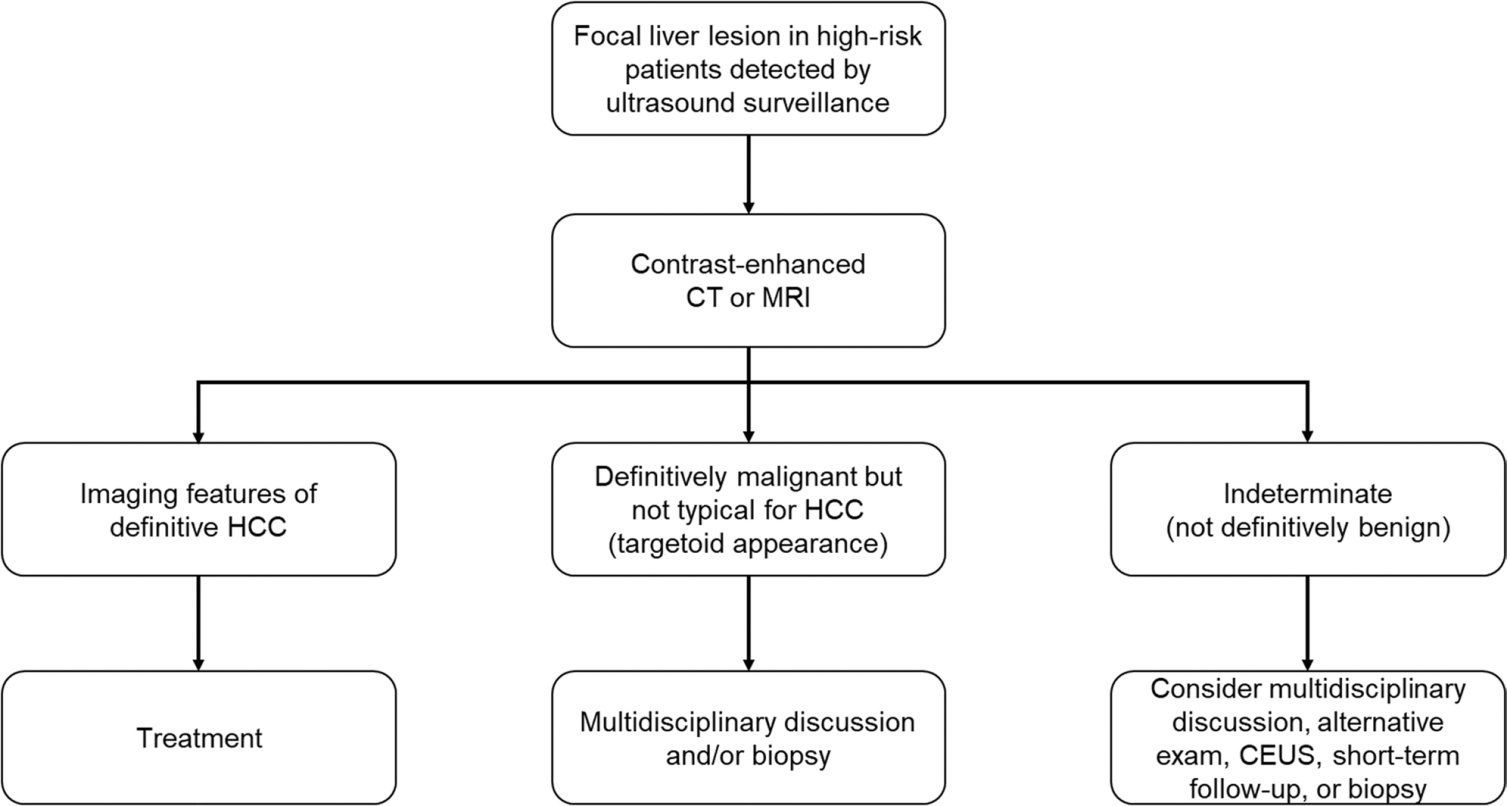
Diagnostic approach of focal liver lesions in high-risk patients

The diagnosis of HCC can be obtained noninvasively without the need of histopathological confirmation in presence of typical imaging features on contrast-enhanced CT (CECT), MRI, or contrast-enhanced ultrasound (CEUS) in patients at high risk (Fig. 1). Several international guidelines are available for the diagnosis of HCC, including the European Association for the Study of the Liver (EASL) [3], the American Association for the Study of Liver Diseases (AASLD) which endorsed the Liver Imaging Reporting and Data System (LI-RADS) algorithm [4–6], the Korean Liver Cancer Association-National Cancer Center (KLCA-NCC) [7], and the Asian-Pacific Association for the Study of the Liver (APASL) [8] guidelines. These guidelines provide a combination of different imaging criteria for the diagnosis of HCC (Table 1) reflecting the geographical differences in HCC risk factors, availability of diagnostic modalities or contrast agents, and treatment strategies.

**Table 1:** Current guidelines for the noninvasive diagnosis of definitive hepatocellular carcinoma (HCC)

	EASL 2018	AASLD/LI-RADS v2018	KLCA-NCC 2022	APASL 2017
Target high-risk patients	Cirrhosis	Cirrhosis, chronic hepatitis B, current or prior HCC history	Cirrhosis, chronic hepatitis B, chronic hepatitis C	Cirrhosis, chronic hepatitis B, chronic hepatitis C
Imaging criteria for definitive HCC on CT/MRI	- Size ≥ 10 mm, APHE and washout	- Non-rim APHE, size ≥ 20 mm, and at least one additional major feature (nonperipheral washout, enhancing capsule, or threshold growth) - Non-rim APHE, size 10–19 mm, and nonperipheral washout or threshold growth - Non-rim APHE, size 10–19 mm, and at least two major features	- Size ≥ 10 mm, APHE and washout on PVP, TP or HBP, without marked T2 hyperintensity or targetoid appearances	- APHE and washout, regardless of the size - APHE and hypointensity on HBP, regardless of the size, after exclusion of hemangioma
Accepted post-arterial phases for the assessment of definitive HCC on MRI with gadoxetate disodium	PVP only	PVP only	PVP, TP, or HBP	PVP or HBP
Imaging criteria for definitive HCC on CEUS	- Size ≥ 10 mm, APHE and late-onset (> 60 s) washout	- Non-rim APHE, size ≥ 10 mm, and late (≥ 60 s) and mild washout	- Size ≥ 10 mm, non-rim APHE, late (≥ 60 s) and mild washout or washout in the Kupffer cell phase	- APHE, washout in vascular phase or hypoechoic the Kupffer cell phase, regardless of the size
Imaging criteria for macrovascular invasion	- APHE and restricted diffusion	- Unequivocal enhancing soft tissue in vein, regardless of visualization of parenchymal mass	–	–
Imaging criteria for non-HCC malignancies	–	- Targetoid mass including rim APHE, peripheral washout, delayed central enhancement, targetoid restriction, targetoid appearance on TP or HBP - Non-targetoid mass (not LR-TIV nor LR-5) with infiltrative appearance, marked diffusion restriction, necrosis or severe ischemia -Early (< 60 s) and/or marked washout on CEUS	- Targetoid appearances on DWI or contrast-enhanced images - Early washout (< 60 s) or punched-out pattern washout within 120 s on CEUS	–

*APHE* arterial phase hyperenhancement, *HBP* hepatobiliary phase, *HCC* hepatocellular carcinoma, *PVP* portal venous phase, *TP* transitional phase

As a part of the “ESR Essentials” series, this article provides a concise and relevant practice recommendations aimed at general radiologist audience in order to summarize the best practice and to inform on the essential imaging criteria for the diagnosis of HCC.

## High-risk patients

Noninvasive diagnostic criteria for the diagnosis of HCC can be applied only in patients with high risk factors in order to maintain a high specificity for the HCC diagnosis. Criteria for defining high-risk patients vary in HCC guidelines due to the different epidemiology and incidence of HCC worldwide. According to EASL guidelines, noninvasive criteria for HCC diagnosis can be applied only to patients with cirrhosis, while the HCC diagnosis in noncirrhotic patients should be confirmed by histopathology [3]. The LI-RADS defines high-risk patients if having cirrhosis, chronic viral hepatitis B (even in absence of cirrhosis), and current or prior HCC history, including adult liver transplant candidates and recipients. LI-RADS criteria cannot be applied to patients with cirrhosis secondary to congenital hepatic fibrosis and vascular liver disorders (e.g., Budd-Chiari syndrome, chronic portal vein occlusion), or patients younger than 18 years old [6]. Patients with chronic hepatitis B and chronic hepatitis C are considered at high risk of HCC, even in absence of cirrhosis, according to the KLCA-NCC and APASL guidelines [7, 8]. Currently, there is insufficient data on the performance of noninvasive imaging criteria for HCC diagnosis in noncirrhotic patients with nonalcoholic fatty liver disease, which cannot be included in the high-risk population when being noncirrhotic.

In patients without the above-defined risk factors, the combination of the typical imaging features of HCC (arterial phase hyperenhancement and washout on portal venous or delayed phases) can be observed in other hepatic lesions such as hypervascular metastases or some hepatocellular adenoma subtypes. Therefore, the HCC diagnosis in patients not meeting the high-risk population criteria should be confirmed by histopathological analysis.

## Diagnostic imaging modalities

Ultrasound is the recommended imaging exam for HCC surveillance in high-risk patients due to its cost-effectiveness and repeatability, but it is operator-dependent, can be limited by several patient-related factors such as obesity and ascites, and has limited sensitivity for the detection of small lesions [9]. Either multiphasic CECT or MRI can be performed for lesion characterization. MRI can be acquired with extracellular or hepatobiliary contrast agents, and it should be the preferred imaging modality for HCC diagnosis due to its higher contrast resolution and sensitivity for the detection of small HCC. Recent meta-analysis demonstrated that CT and MRI have similar specificity for the diagnosis of HCC (both above 90% in high-risk patients), but MRI provides a higher sensitivity compared to CT (61–82% vs 48–66%) [10, 11]. However, the choice of the diagnostic exam and contrast agent should take into account the local availability, radiologist expertise, and patient characteristics including contraindications to specific modality (for example, radiation exposure in young patients with chronic liver disease for CT, incompatible devices, claustrophobia caused by MRI) or contrast agent. Recent intraindividual studies comparing gadolinium-based contrast agents demonstrated higher diagnostic performances of MRI acquired with extracellular contrast agents compared to the MRI acquired with gadoxetate disodium according to current LI-RADS and EASL criteria for the noninvasive diagnosis of HCC [12–14].

CEUS can be adopted as a problem-solving technique in patients with inconclusive multiphasic cross-sectional imaging in experienced centers. CEUS has the advantage to provide higher temporal resolution compared to cross-sectional exams with the possibility to evaluate the entire arterial phase. However, CEUS can evaluate single lesion and it has limitations related to operator experience, patient factors, and deep tumor location.

Adequate imaging technique is crucial for the diagnosis of HCC. CECT should be acquired with multi-detector row scanners, including at least the late hepatic arterial phase acquired with the bolus tracking methods at about 35–45 s after the contrast agent injection, portal venous (60–75 s), and delayed (3–5 min) phases after the intravenous injection of iodinate contrast agent at the 3–5 ml/s injection rate [15]. MRI exams should be acquired with 1.5-T or higher field scanners, equipped with a phased-array multichannel surface coil and include at least the following sequences: T2-weighted images with and without fat saturation, T1-weighted in-phase and opposed-phase images, diffusion-weighted imaging with at least one acquisition with *b*-value of 400–800 s/mm^2^, pre-contrast and post-contrast 3D T1-weighted images on late hepatic arterial phase acquired with bolus detection technique, portal venous, delayed (2–5 min with extracellular contrast agents or gadobenate dimeglumine), or transitional (with gadoxetate disodium) phases [16]. Hepatobiliary phase images should be acquired at 20 min after the administration of gadodexate disodium and at 1–3 h after the administration of gadobenate dimeglumine. Slice thickness of 5 mm or less is recommended for dynamic imaging [15].

## Imaging features

### Size

Size of malignant hepatocellular nodules progressively increases during the hepatocarcinogenesis process. Current clinical guidelines differ in the adoption of the size-based pathway or the non-size-based pathway. In the EASL, LI-RADS, and KLCA-NCC, a diameter equal or larger than 10 mm is required for the diagnosis of definitive HCC, while in the APASL guideline HCC can be noninvasively diagnosed in any tumor size in presence of typical imaging features [3, 6–8]. Size affects the clinical staging and treatment recommendations in patients with HCC. Solitary HCCs measuring ≤ 2 cm are considered very early stage in the Barcelona Clinic Liver Cancer (BCLC) system and resection or ablation is the recommended treatment [3].

Lesion size is defined as the largest diameter measured from the outer edge to the outer edge on the longest axis, including the capsule if present. Size should be measured in the phase or sequence in which the lesion is better demarcated. Arterial phase and diffusion-weighted imaging should be avoided when the lesion can be measured in other phases, as peritumoral perfusion alteration and lower resolution can impact the size measurement.

### Arterial phase hyperenhancement

**Fig. 2:**
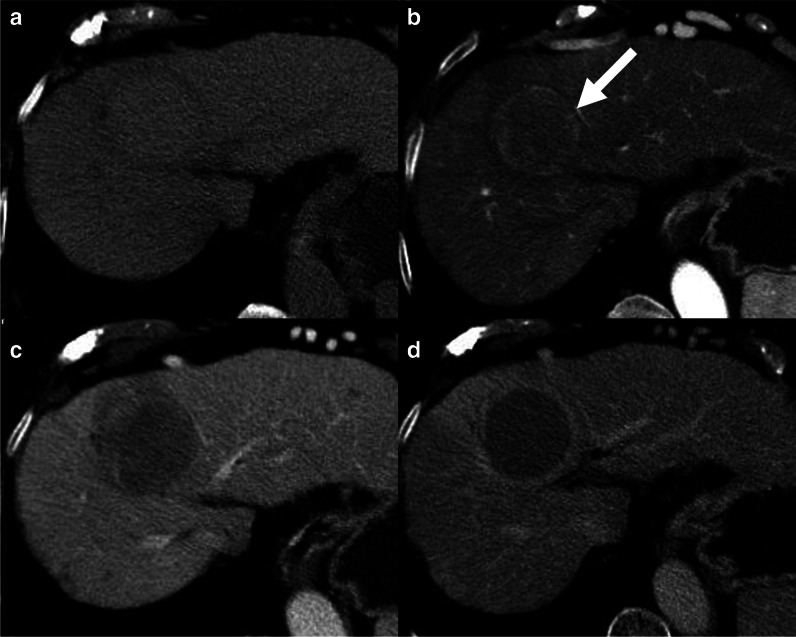
A 75-year-old woman with cirrhosis and hepatocellular carcinoma. Contrast-enhanced CT shows a lesion isodense on pre-contrast (**a**), with non-rim arterial phase hyperenhancement (**b**, arrow), non-peripheral washout on portal venous (**c**) and delayed (**d**) phases. Enhancing capsule is also visible on the delayed phase

Arterial phase hyperenhancement (APHE) is an imaging feature strongly associated with the diagnosis of HCC and all the guidelines concord that it is necessary to reach the diagnosis of HCC on imaging [17]. APHE is defined by the unequivocal hypervascularity on the late hepatic arterial phase compared to the background liver parenchyma (Fig. 2) [6]. This imaging feature is the expression of the progressive neoangiogenesis occurring in the HCC hepatocarcinogenesis process, in which the normal portal venous supply is replaced by the development of intratumoral impaired arteries. It is important to note that an optimal late hepatic arterial phase should be acquired, in which the contrast has reached the portal vein but not the hepatic veins. APHE may be missed if the arterial phase is acquired too early (the portal vein is not yet enhanced) or too late (the hepatic veins are already enhanced). On MRI, subtraction images should be used to increase the sensitivity for the detection of APHE in lesions with hyperintensity on the pre-contrast T1-weighted images. Multi-arterial phase can improve the quality of adequate arterial phase and APHE detection rate compared to single arterial phase.

It is important to differentiate non-rim APHE from rim APHE. Typical HCCs demonstrate non-rim APHE which is defined as increased enhancement compared to the background parenchyma, visualized in whole or part of the lesion but not at the periphery [6]. Non-rim APHE has a sensitivity of 65–85% for the diagnosis of HCC but a limited specificity of 57% as standalone feature [18, 19]. Up to 40% of HCC may lack APHE, more commonly in the very early HCCs or less differentiated HCCs [20]. Lesions other than HCC may demonstrate non-rim APHE in high-risk patients including dysplastic nodules, capillary hemangiomas, transient hepatic attenuation/intensity differences (THADs/THIDs), and small intrahepatic cholangiocarcinomas. Therefore, non-rim APHE should be combined with other major features for the definitive diagnosis of HCC.

Rim APHE is characterized by an enhancement more prominent at the periphery of the lesion. Rim APHE is an imaging feature suggesting the diagnosis of a non-HCC malignancy such as intrahepatic cholangiocarcinoma, combined hepatocellular-cholangiocarcinoma, or occasionally metastasis, but it may also be observed in atypical HCCs [21]. When using the LI-RADS algorithm, lesions with targetoid appearance, including targetoid dynamic enhancement such as rim APHE, should be classified as LR-M and a histopathological diagnosis is often required for the definitive diagnosis [6].

### Washout

Washout is defined as a temporal reduction of the density/signal intensity of the lesion in the extracellular post-arterial phases compared to the arterial phase, with the lesion being hypodense/hypointense compared to the background liver parenchyma [18]. This feature is attributed to the loss of portal venous supply in progressed HCC. Non-peripheral washout is a major feature associated with the diagnosis of HCC and it can be visualized in whole or part of the lesion, and it can be heterogeneous depending on the different tumoral components [6, 17]. Non-peripheral washout has a sensitivity of 77% and a specificity of 74% for the diagnosis of HCC as standalone feature but it can also be observed in dysplastic nodules and other non-HCC malignancies in high-risk patients [19]. Peripheral washout, defined as washout more pronounced at the periphery of the lesion, is an imaging feature associated with the diagnosis of non-HCC malignancies, and it may be used to categorize an observation as LR-M in the LI-RADS algorithm [6].

**Fig. 3:**
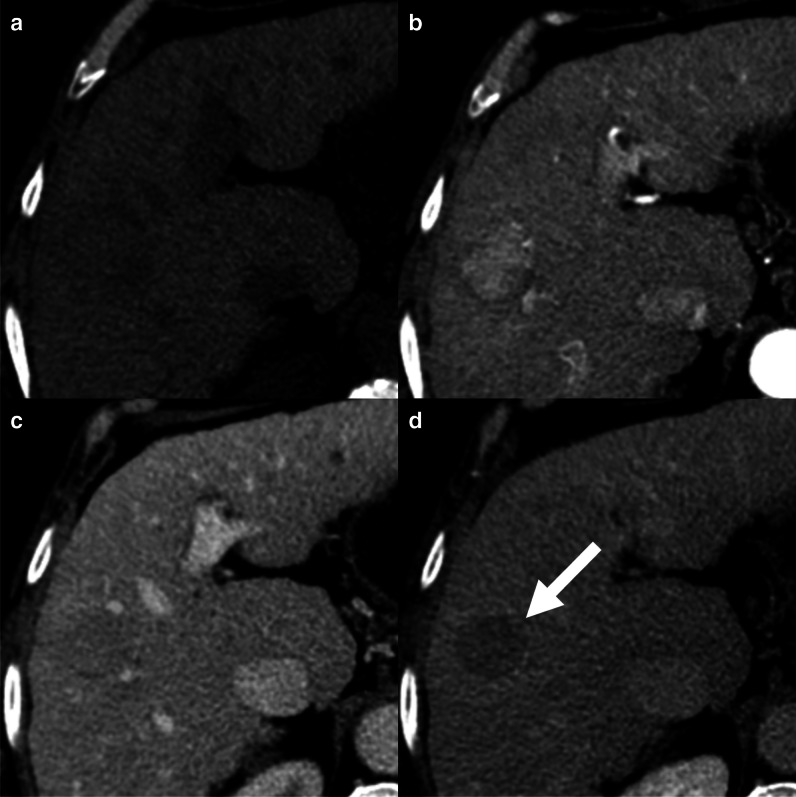
A 66-year-old woman with cirrhosis and hepatocellular carcinoma. Contrast-enhanced CT shows a lesion isodense on pre-contrast (**a**), with non-rim arterial phase hyperenhancement (**b**), isodense on portal venous phase (**c**) but with non-peripheral washout on delayed phase (**d**)

On CECT and MRI acquired with the administration of extracellular contrast agents or gadobenate dimeglumine, all the guidelines are concordant that washout can be evaluated on either portal venous or delayed phases. About 30% of HCCs demonstrate washout during the delayed phase only (Fig. 3); therefore, acquiring a delayed phase is mandatory in the HCC imaging protocol [22]. Washout should be evaluated on portal venous phase only when MRI is acquired with the administration of gadoxetate disodium. The uptake of gadoxetate by non-tumoral hepatocytes starts at about 90 s after contrast injection [18]. Images acquired at 2–5 min (named as transitional phases) reflect a combination of early hepatocyte uptake and extracellular contrast distribution. Several lesions other than HCC demonstrate hypointensity on transitional phase, including hemangiomas, dysplastic nodules, and non-HCC malignancies, reducing the specificity for the diagnosis of HCC [23]. In the LI-RADS algorithm, transitional phase hypointensity is considered an ancillary feature favoring malignancy [6], while the KLCA-NCC guidelines allow the assessment of washout on transitional phase in lesions without marked T2 hyperintensity or targetoid appearances [7].

### Capsule

**Fig. 4:**
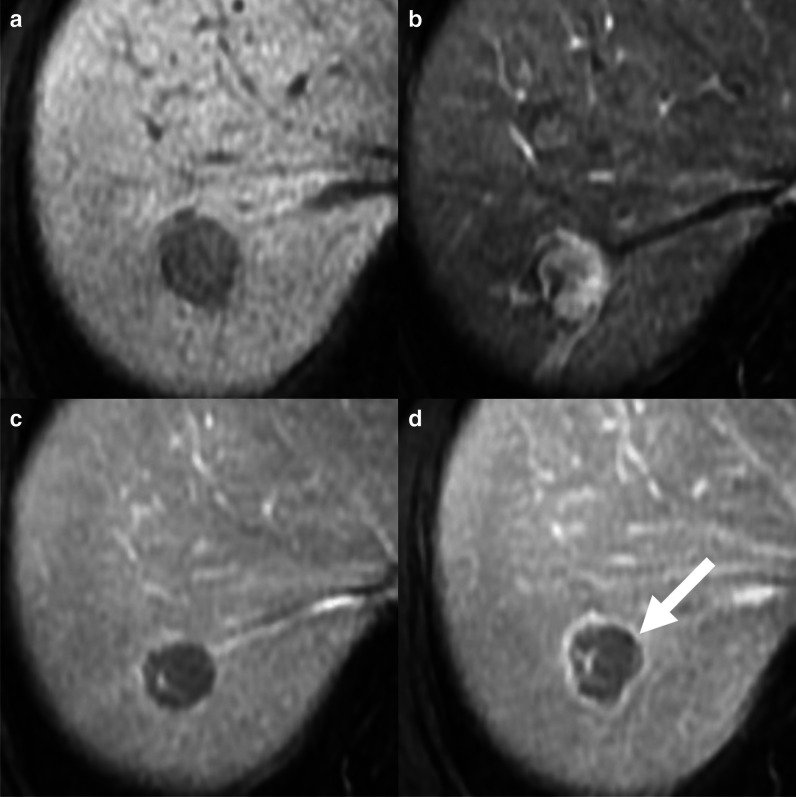
A 66-year-old man with cirrhosis and hepatocellular carcinoma. MRI acquired after the administration of extracellular contrast agent shows a 20-mm lesion with hypointensity on pre-contrast image (**a**), non-rim arterial phase hyperenhancement (**b**), non-peripheral washout and enhancing capsule on portal venous (**c**) and delayed (**d**, arrow) phases

Pathological tumor capsule is a typical feature of progressed HCC, and it reflects the combination of peritumoral fibrous tissue, prominent sinusoid, and perilesional compressed liver parenchyma. Tumor capsule is rarely observed in small or early HCCs as well as in poorly differentiated or infiltrative lesions. Two types of capsule appearance can be observed on imaging. The enhancing capsule is defined as a smooth border surrounding part of the whole lesion, more conspicuous than fibrotic tissue in the liver parenchyma, appearing as an enhancing rim in the portal venous, delayed, or transitional phases [6]. The enhancing capsule can be detected on imaging in about 50% of HCCs, being more frequently observed on MRI with extracellular contrast agents (Fig. 4) compared to CECT or MRI with gadoxetate disodium, while it cannot be depicted on CEUS due to the use of purely intravascular contrast agents [24, 25]. The enhancing capsule detected on imaging provides a sensitivity of 52% and a specificity of 90% for the diagnosis of HCC [19].

The non-enhancing capsule is another subtype of the capsule appearance that can be identified as a non-enhancing peripheral rim on pre-contrast and T2-weighted sequences, or a hypointense rim on the hepatobiliary phase.

The enhancing capsule is considered a major feature for the diagnosis of HCC only in the CT/MRI LI-RADS v2018 algorithm, while the non-enhancing capsule is included in the ancillary features favoring malignancy, HCC in particular [6]. Enhancing and non-enhancing capsule are included in the ancillary features favoring the diagnosis of HCC in particular in the KLCA-NCC guideline [7].

### Growth

Tumor growth is an imaging feature suggesting malignancy related to the progressive cell duplication in the mass. The estimated median doubling time for HCC has been reported to be 178 days, but it can vary greatly according to the tumor size, histopathological grade, and vascular invasion [18]. Tumor growth is not a unique feature of HCC, and it can be observed in all malignant lesions and pre-malignant nodules. Therefore, this imaging feature should be always considered in combination with other major features of HCC.

The LI-RADS v2018 algorithm considers threshold growth a major feature for the diagnosis of HCC, and it is defined as an unequivocal increase in size of a mass ≥ 50% in less than 6 months [6]. Unequivocal growth < 50%, growth in more than 6 months, or newly appearing lesion in any interval time, should be categorized as sub-threshold growth, an ancillary feature favoring malignancy, not HCC in particular [6]. Importantly, growth should by applied only if a prior CT or MRI are available with sufficient quality to measure the lesion, while it cannot be applied to prior ultrasound or CEUS exams. Tumor size should be compared in the images acquired in the same phase and plane.

### Hepatobiliary phase hypointensity

**Fig. 5:**
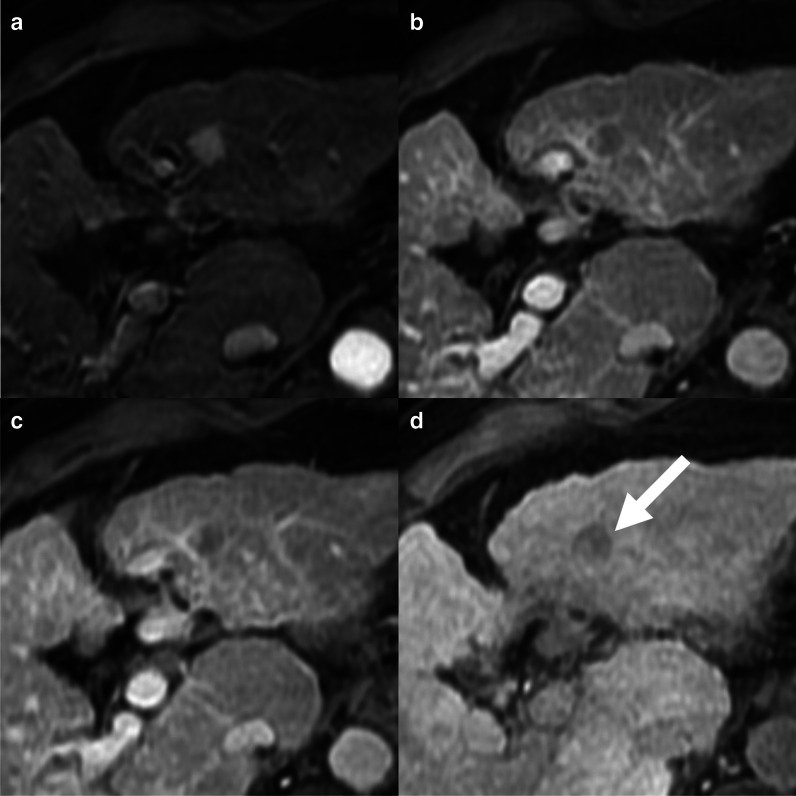
A 69-year-old man with cirrhosis and hepatocellular carcinoma. MRI acquired after the administration of gadoxetate disodium shows a 10-mm lesion with non-rim arterial phase hyperenhancement (**a**), non-peripheral washout and enhancing capsule on portal venous (**b**) and 3-min transitional (**c**) phases, and hypointensity on the 20-min hepatobiliary phase (**d**, arrow)

Hepatobiliary phase hypointensity refers to an unequivocal lower intensity in part or the whole lesion compared to the background liver parenchyma in the hepatobiliary phase acquired after the administration of gadoxetate disodium or gadobenate dimeglumine (Fig. 5). The hepatobiliary phase hypointensity is related to the progressively reduced expression of the organic anion transporting polypeptide (OATP) 1B3, which is responsible for the contrast agent uptake by the normal hepatocytes [26]. This imaging feature should be evaluated only if the hepatobiliary phase has an adequate quality, with hepatic vessels being definitely hypointense in comparison to the background liver parenchyma and with contrast agent being detected in the intrahepatic bile ducts [27]. In patients with suboptimal hepatobiliary phase due to poor liver function, the assessment of this feature is unreliable.

HCC demonstrates hepatobiliary phase hypointensity in about 90% of cases, while about 5–10% of HCCs are isointense or hyperintense on the hepatobiliary phase due to their higher OATP 1B3 expression [26]. Hepatobiliary phase hypointensity increases the sensitivity for the diagnosis of HCC, particularly in lesions lacking washout on portal venous phase [28]. In patients at high risk of HCC, hypointense nodules on hepatobiliary phase without APHE have been proven to be progressed HCC, early HCC, or high-grade dysplastic nodule at pathology in more than 90% of cases [29]. However, hepatobiliary phase hypointensity is not a specific feature of HCC and it can be observed in other benign lesions, such as hemangiomas, confluent fibrosis, some dysplastic nodules, some perfusion alteration, and in non-HCC malignancies. Using hepatobiliary phase as an alternative to washout leads to a significant decrease in specificity for the diagnosis of HCC [23, 29].

The hepatobiliary phase hypointensity is a major imaging feature for the diagnosis of HCC according to the KLCA-NCC and APASL guidelines but it is included in the ancillary features favoring malignancy, not HCC in particular, in the LI-RADS v2018 algorithm [6–8]. A targetoid appearance on the hepatobiliary phase should suggest the diagnosis of non-HCC malignancy.

### Macrovascular invasion

**Fig. 6:**
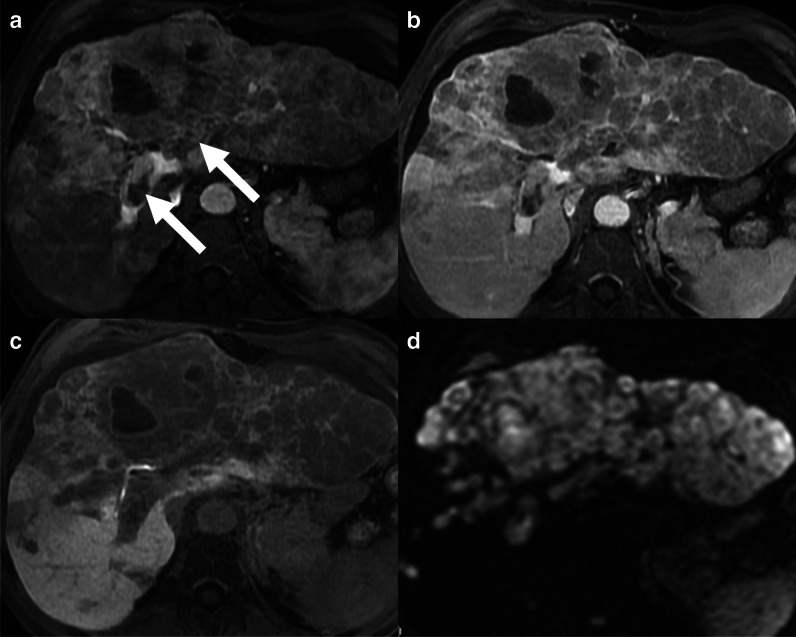
A 58-year-old man with cirrhosis and infiltrative hepatocellular carcinoma with macrovascular invasion. MRI acquired after the administration of gadoxetate disodium shows a bilobar infiltrative lesion with non-rim arterial phase hyperenhancement and macrovascular invasion in the left and right portal vein branches (**a**, arrows), non-peripheral washout on portal venous (**b**), hypointensity on the 20-min hepatobiliary phase (**c**), and marked restricted diffusion (**d**)

Advanced HCC may progress to macrovascular invasion with malignant tumoral thrombosis in the portal vein or more rarely in the hepatic veins. Macrovascular invasion is a poor prognostic factor of HCC, associated with shorter survival, and it is a contraindication for liver transplantation [3]. Patients with advanced chronic liver disease may also develop non-tumoral portal vein thrombosis as a complication. The differential diagnosis between benign and malignant portal vein thrombosis is crucial for the management of patients with HCC. The most important imaging feature for the diagnosis of tumoral portal vein thrombosis is the visualization of unequivocally enhancing soft tissues in the vein (Fig. 6), providing a sensitivity of 64% and a specificity of 99.8% for the diagnosis of macrovascular invasion [30]. Other imaging features that may suggest the diagnosis of tumor in vein are the increased diameter of the portal vein, occluded vein with ill-defined margins, restricted diffusion in the thrombus, and contiguity with a parenchymal lesion [6]. Advanced HCCs may also present as an infiltrative mass characterized by the spread of multiple ill-defined nodules in the liver parenchyma, reflecting the diffuse infiltration of the liver parenchyma by tumoral nodules, often associated with the macrovascular invasion.

Macrovascular invasion is not exclusive to HCC, and it can be observed in other non-HCC malignancies including combined hepatocellular-cholangiocarcinoma and occasionally in intrahepatic cholangiocarcinoma. LI-RADS categorizes lesions with definitive macrovascular invasion as LR-TIV (tumor in vein) with the recommendation to report the most probable etiology of the tumor thrombus.

## Summary statement

HCC can be diagnosed noninvasively in the presence of typical imaging features on contrast-enhanced exams, including size, non-rim arterial phase hyperenhancement, non-peripheral washout, enhancing capsule, and growth. These criteria should be applied only in patients at high risk, with an elevated pre-test probability of having an HCC. Most of the guidelines are concordant that lesion size of at least 10 mm, non-rim arterial phase hyperenhancement, and non-peripheral washout are required to diagnose definitive HCC without needing histopathological confirmation. CECT and MRI with extracellular or hepatobiliary contrast agents are the recommended techniques for the diagnosis of HCC, and the choice of imaging exams and type of contrast agent should consider the availability, expertise, and patient characteristics. CEUS can be used as a problem-solving technique in atypical lesions or in patients with contraindications to CT/MRI contrast agents. Targetoid imaging features, including rim arterial phase hyperenhancement, peripheral washout, targetoid restriction, or targetoid appearance on transitional or hepatobiliary phase, are not typical of HCC and the diagnosis of non-HCC malignancy should be considered. Macrovascular invasion can be diagnosed in the presence of unequivocal enhancing soft tissue in the venous thrombus.

## Patient summary

The diagnosis of hepatocellular carcinoma can be made on different imaging exams acquired with the intravenous administration of contrast agents, including computed tomography (CT), magnetic resonance imaging (MRI), or sometimes contrast-enhanced ultrasound (CEUS). In patients with liver cirrhosis or other high risk factors, hepatic lesions with typical imaging characteristics can be diagnosed as hepatocellular carcinoma without the need of other exams. Hepatic lesions with atypical imaging characteristics may require additional imaging exams, follow-up over time, or histopathological analysis with biopsy of the lesion. The management of hepatocellular carcinoma is often discussed at multidisciplinary meetings to choose the most appropriate treatment.

## Abbreviations

APASL: Asian-Pacific Association for the Study of the Liver
APHE: Arterial phase hyperenhancement
CECT: Contrast-enhanced CT
CEUS: Contrast-enhanced ultrasound
EASL: European Association for the Study of the Liver
HCC: Hepatocellular carcinoma
KLCA-NCC: Korean Liver Cancer Association-National Cancer Center
LI-RADS: Liver Imaging Reporting and Data System

## Declarations

### Guarantor

The scientific guarantor of this publication is Giuseppe Brancatelli.

### Statistics and biometry

No complex statistical methods were necessary for this paper.

### Methodology

• Practice recommendations

## References

[1] Sung H, Ferlay J, Siegel RL et al (2021) Global Cancer Statistics 2020: GLOBOCAN Estimates of Incidence and Mortality Worldwide for 36 Cancers in 185 Countries. CA Cancer J Clin 71:209–249. 10.3322/caac.21660 33538338 10.3322/caac.21660

[2] McGlynn KA, Petrick JL, El-Serag HB (2021) Epidemiology of hepatocellular carcinoma. Hepatology 73(Suppl 1):4–13. 10.1002/hep.31288 32319693 10.1002/hep.31288 PMC7577946

[3] European Association for the Study of the Liver (2018) EASL Clinical Practice Guidelines: Management of hepatocellular carcinoma. J Hepatol 69:182–236. 10.1016/j.jhep.2018.03.019 29628281 10.1016/j.jhep.2018.03.019

[4] Singal AG, Llovet JM, Yarchoan M et al (2023) AASLD practice guidance on prevention, diagnosis, and treatment of hepatocellular carcinoma. Hepatology. 10.1097/HEP.0000000000000466 37199193 10.1097/HEP.0000000000000466 PMC10663390

[5] Marrero JA, Kulik LM, Sirlin CB et al (2018) Diagnosis, staging, and management of hepatocellular carcinoma: 2018 Practice Guidance by the American Association for the Study of Liver Diseases. Hepatology 68:723–750. 10.1002/hep.29913 29624699 10.1002/hep.29913

[6] American College of Radiology. CT/MRI Liver imaging reporting and data system v2018 core. https://www.acr.org/-/media/ACR/Files/RADS/LI-RADS/LI-RADS-2018-Core.pdf. Accessed July 2023

[7] Korean Liver Cancer Association (KLCA) and National Cancer Center (NCC) Korea (2022) 2022 KLCA-NCC Korea practice guidelines for the management of hepatocellular carcinoma. Clin Mol Hepatol 28:583–705. 10.3350/cmh.2022.0294 36263666 10.3350/cmh.2022.0294 PMC9597235

[8] Omata M, Cheng AL, Kokudo N et al (2017) Asia-Pacific clinical practice guidelines on the management of hepatocellular carcinoma: a 2017 update. Hepatol Int 11:317–370. 10.1007/s12072-017-9799-9 28620797 10.1007/s12072-017-9799-9 PMC5491694

[9] Wen N, Cai Y, Li F et al (2022) The clinical management of hepatocellular carcinoma worldwide: a concise review and comparison of current guidelines: 2022 update. Biosci Trends 16:20–30. 10.5582/bst.2022.01061 35197399 10.5582/bst.2022.01061

[10] Roberts LR, Sirlin CB, Zaiem F et al (2018) Imaging for the diagnosis of hepatocellular carcinoma: a systematic review and meta-analysis. Hepatology 67:401–421. 10.1002/hep.29487 28859233 10.1002/hep.29487

[11] Kim YY, Lee S, Shin J et al (2022) Diagnostic performance of CT versus MRI Liver Imaging Reporting and Data System category 5 for hepatocellular carcinoma: a systematic review and meta-analysis of comparative studies. Eur Radiol 32:6723–6729. 10.1007/s00330-022-08985-z 35849177 10.1007/s00330-022-08985-z

[12] Min JH, Kim JM, Kim YK et al (2018) Prospective intraindividual comparison of magnetic resonance imaging with gadoxetic acid and extracellular contrast for diagnosis of hepatocellular carcinomas using the Liver Imaging Reporting and Data System. Hepatology 68:2254–2266. 10.1002/hep.30122 30070365 10.1002/hep.30122

[13] Paisant A, Vilgrain V, Riou J et al (2020) Comparison of extracellular and hepatobiliary MR contrast agents for the diagnosis of small HCCs. J Hepatol 72:937–945. 10.1016/j.jhep.2019.12.011 31870951 10.1016/j.jhep.2019.12.011

[14] Rimola J, Sapena V, Brancatelli G et al (2022) Reliability of extracellular contrast versus gadoxetic acid in assessing small liver lesions using liver imaging reporting and data system vol 2018 and European association for the study of the liver criteria. Hepatology 76:1318–1328. 10.1002/hep.32494 35349760 10.1002/hep.32494

[15] Kambadakone AR, Fung A, Gupta RT et al (2018) LI-RADS technical requirements for CT, MRI, and contrast-enhanced ultrasound. Abdom Radiol (NY) 43:56–74. 10.1007/s00261-017-1325-y 28940042 10.1007/s00261-017-1325-y

[16] ACR–SAR–SPR practice parameter for the performance of magnetic resonance imaging (MRI) of the liver. Available at https://www.acr.org/-/media/ACR/Files/Practice-Parameters/MR-Liver.pdf. Accessed July 2023

[17] van der Pol CB, McInnes MDF, Salameh JP et al (2022) CT/MRI and CEUS LI-RADS major features association with hepatocellular carcinoma: individual patient data meta-analysis. Radiology 302:326–335. 10.1148/radiol.2021211244 34783596 10.1148/radiol.2021211244

[18] Tang A, Bashir MR, Corwin MT et al (2018) Evidence supporting LI-RADS major features for CT- and MR imaging-based diagnosis of hepatocellular carcinoma: a systematic review. Radiology 286:29–48. 10.1148/radiol.2017170554 29166245 10.1148/radiol.2017170554 PMC6677284

[19] Shin J, Lee S, Yoon JK, Chung YE, Choi JY, Park MS (2021) LI-RADS major features on MRI for diagnosing hepatocellular carcinoma: a systematic review and meta-analysis. J Magn Reson Imaging 54:518–525. 10.1002/jmri.27570 33638582 10.1002/jmri.27570

[20] Ronot M, Purcell Y, Vilgrain V (2019) Hepatocellular carcinoma: current imaging modalities for diagnosis and prognosis. Dig Dis Sci 64:934–950. 10.1007/s10620-019-05547-0 30825108 10.1007/s10620-019-05547-0

[21] Shin J, Lee S, Hwang JA et al (2022) MRI-diagnosis of category LR-M observations in the Liver Imaging Reporting and Data System v2018: a systematic review and meta-analysis. Eur Radiol 32:3319–3326. 10.1007/s00330-021-08382-y 35031839 10.1007/s00330-021-08382-y

[22] Furlan A, Marin D, Vanzulli A et al (2011) Hepatocellular carcinoma in cirrhotic patients at multidetector CT: hepatic venous phase versus delayed phase for the detection of tumour washout. Br J Radiol 84:403–412. 10.1259/bjr/18329080 21081569 10.1259/bjr/18329080 PMC3473662

[23] Pan J, Tao Y, Chi X, Yang L, Zhao Y, Chen F (2022) Do transition and hepatobiliary phase hypointensity improve LI-RADS categorization as an alternative washout: a systematic review and meta-analysis. Eur Radiol 32:5134–5143. 10.1007/s00330-022-08665-y 35267090 10.1007/s00330-022-08665-y

[24] Dioguardi Burgio M, Picone D, Cabibbo G, Midiri M, Lagalla R, Brancatelli G (2016) MR-imaging features of hepatocellular carcinoma capsule appearance in cirrhotic liver: comparison of gadoxetic acid and gadobenate dimeglumine. Abdom Radiol (NY) 41:1546–1554. 10.1007/s00261-016-0726-7 27052455 10.1007/s00261-016-0726-7

[25] Cannella R, Ronot M, Sartoris R et al (2021) Enhancing capsule in hepatocellular carcinoma: intra-individual comparison between CT and MRI with extracellular contrast agent. Diagn Interv Imaging 102:735–742. 10.1016/j.diii.2021.06.004 34284951 10.1016/j.diii.2021.06.004

[26] Kitao A, Zen Y, Matsui O et al (2010) Hepatocellular carcinoma: signal intensity at gadoxetic acid-enhanced MR imaging–correlation with molecular transporters and histopathologic features. Radiology 256:817–826. 10.1148/radiol.10092214 20663969 10.1148/radiol.10092214

[27] Neri E, Bali MA, Ba-Ssalamah A et al (2016) ESGAR consensus statement on liver MR imaging and clinical use of liver-specific contrast agents. Eur Radiol 26:921–931. 10.1007/s00330-015-3900-3 26194455 10.1007/s00330-015-3900-3 PMC4778143

[28] Joo I, Lee JM, Lee DH, Jeon JH, Han JK (2019) Retrospective validation of a new diagnostic criterion for hepatocellular carcinoma on gadoxetic acid-enhanced MRI: can hypointensity on the hepatobiliary phase be used as an alternative to washout with the aid of ancillary features? Eur Radiol 29:1724–1732. 10.1007/s00330-018-5727-1 30255250 10.1007/s00330-018-5727-1

[29] Joo I, Kim SY, Kang TW et al (2020) Radiologic-pathologic correlation of hepatobiliary phase hypointense nodules without arterial phase hyperenhancement at gadoxetic acid-enhanced MRI: a multicenter study. Radiology 296:335–345. 10.1148/radiol.2020192275 32484414 10.1148/radiol.2020192275

[30] Bae JS, Lee JM, Jeon SK et al (2022) LI-RADS tumor in vein at CT and hepatobiliary MRI. Radiology 302:107–115. 10.1148/radiol.2021210215 34581625 10.1148/radiol.2021210215

